# Getting to the heart of intracellular glucocorticoid regeneration: 11β-HSD1 in the myocardium

**DOI:** 10.1530/JME-16-0128

**Published:** 2016-12-03

**Authors:** Gillian A Gray, Christopher I White, Raphael F P Castellan, Sara J McSweeney, Karen E Chapman

**Affiliations:** University/BHF Centre for Cardiovascular ScienceQueen’s Medical Research Institute, University of Edinburgh, Edinburgh, UK

**Keywords:** 11β-HSD1, 11β-HSD2, H6PDH, 11β-HSD1 inhibitor, cardiomyocyte, fibroblast, macrophage, myocardial infarction, heart failure

## Abstract

Corticosteroids influence the development and function of the heart and its response to injury and pressure overload via actions on glucocorticoid (GR) and mineralocorticoid (MR) receptors. Systemic corticosteroid concentration depends largely on the activity of the hypothalamic–pituitary–adrenal (HPA) axis, but glucocorticoid can also be regenerated from intrinsically inert metabolites by the enzyme 11β-hydroxysteroid dehydrogenase type 1 (11β-HSD1), selectively increasing glucocorticoid levels within cells and tissues. Extensive studies have revealed the roles for glucocorticoid regeneration by 11β-HSD1 in liver, adipose, brain and other tissues, but until recently, there has been little focus on the heart. This article reviews the evidence for glucocorticoid metabolism by 11β-HSD1 in the heart and for a role of 11β-HSD1 activity in determining the myocardial growth and physiological function. We also consider the potential of 11β-HSD1 as a therapeutic target to enhance repair after myocardial infarction and to prevent the development of cardiac remodelling and heart failure.

## Introduction

The major physiological adrenocorticosteroid hor­mones, glucocorticoids (cortisol in most animals and corticosterone in rats and mice) and mineralocorticoids (aldosterone) are vital for normal cardiovascular function. They regulate blood pressure (Hunter & Bailey 2015) and vascular tone (Ullian 1999, Dover *et al.* 2007, Hadoke *et al.* 2009), as well as heart rhythm and contractility (Lefer 1967, 1968, Penefsky & Kahn 1971, Ouvrard-Pascaud *et al.* 2005, Cruz-Topete *et al.* 2016). Under pathological conditions, rapid corticosteroid release in response to hypothalamic–pituitary–adrenal (HPA) axis activation is an early response to cardiovascular insult, including after myocardial infarction (MI). In the acute period, post-MI corticosteroids are cardioprotective and suppress the early inflammatory response to injury (Libby *et al.* 1973, Skyschally *et al.* 2004). However, sustained excessive glucocorticoid release from the adrenals, for example in Cushing’s syndrome, is associated with detrimental cardiac outcomes, including myocardial ischaemia and hypertrophy, and in rare cases, with loss of cardiomyocytes and dilated cardiomyopathy (Peppa *et al.* 2009, Shibusawa *et al.* 2015, Frustaci *et al.* 2016). Chronic stress associates with cardiovascular disease (Kumari *et al.* 2011), as does pharmacological glucocorticoid treatment (Wei *et al.* 2004, Souverein *et al.* 2004).

Corticosteroids influence cell behaviour primarily through the intracellular activation of glucocorticoid (GR) and mineralocorticoid (MR) receptors. In the heart, activation of both GR and MR has an impact on cardiac development, physiology and pathophysiology (reviewed in Oakley & Cidlowski 2015 and Richardson *et al.* 2016). GR and MR are highly related and belong to the nuclear receptor family of transcriptional regulators (Biddie *et al.* 2010), although some corticosteroid actions may be mediated by non-classical signalling through cell-surface receptors (Mihailidou & Funder 2005, Samarasinghe *et al.* 2012). The GR binds glucocorticoids with 10- to 30-fold lower affinity than the MR, but is relatively selective for glucocorticoid compared with mineralocorticoid binding (Mihailidou & Funder 2005, Samarasinghe *et al.* 2012). The MR has high affinity for both mineralocorticoids and glucocorticoids, but as glucocorticoids typically circulate at levels 100-fold higher than those of mineralocorticoids, the MR is likely to be constitutively occupied by glucocorticoids even at daily nadir levels. Downstream signalling may differ depending on which ligand occupies the receptor and, depending on prevailing conditions, glucocorticoids can behave as agonists or antagonists at the MR (reviewed in Funder 2012). Access of mineralocorticoids to the MR depends on specific mechanisms that regulate the intracellular availability of adrenocorticosteroid hormones (Funder *et al.* 1988). These include the extent of protein binding in the plasma and in the heart itself (Bolton *et al.* 2014, Schafer *et al.* 2015) and the activity of transporters that actively extrude steroids from the cell. However, glucocorticoids also undergo intracellular metabolism, and the capacity of individual cells to metabolise glucocorticoids is a critical factor in determining the extent (and indeed selectivity) of MR and GR activation (Funder *et al.* 1988, Odermatt & Kratschmar 2012, Chapman *et al.* 2013*a*).

## 11β-HSD enzymes

**Figure 1:**
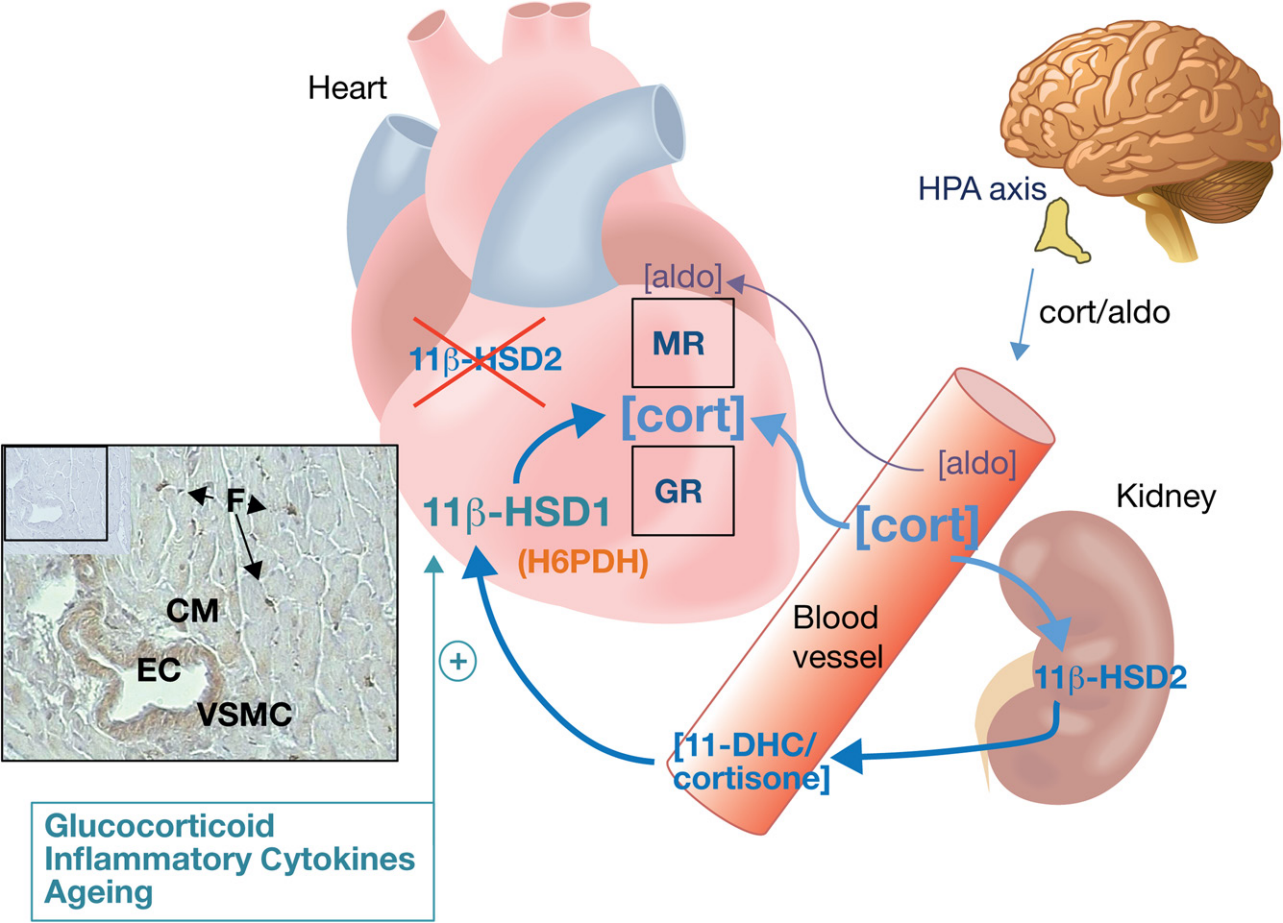
Adrenocorticosteroids, 11β-HSD1 and the heart. Hypothalamic-pituitary-adrenal (HPA) axis-derived glucocorticoids (cortisol in man, corticosterone in rats and mice) and the mineralocorticoid - aldosterone - compete for binding to cardiac glucocorticoid (GR) and mineralocorticoid receptors (MR). Glucocorticoids are also regenerated within the heart from circulating inert metabolites (cortisone in man and 11-dehydrocorticosterone (11-DHC) in rats and mice) by 11β-hydroxysteroid dehydrogenase type 1 (11β-HSD1), when it is expressed alongside hexose-6-phosphate dehydrogenase (H6PDH). As the glucocorticoid concentration ([cort]) normally far exceeds that of aldosterone ([aldo]), GR and MR are usually occupied by glucocorticoids. 11β-hydroxysteroid dehydrogenase type 2 (11β-HSD2) inactivates glucocorticoids, but unlike other MR target tissues, there is normally little or no dehydrogenase activity in the heart. Inactivation of glucocorticoids by 11β-HSD2 activity elsewhere in the body, largely in the kidney, generates the inert precursors that, on entering the circulation, become available for glucocorticoid regeneration and GR/MR activation in cells that express 11β-HSD1. In the healthy heart, 11β-HSD1 immunoreactivity (inset panel) is localised to vascular smooth muscle (VSM), fibroblasts (F) and also cardiomyocytes (CM) in a fixed section from mouse left ventricle. The 11β-HSD1 antibody is a sheep anti-mouse polyclonal generated in house (De Sousa Peixoto *et al*. 2008). Staining is notably absent in endothelial cells (EC) lining the vascular wall. A negative control of the same section generated using sheep IgG is shown in the inset panel. From S J McSweeney, PhD thesis, 2010 (McSweeney 2010). 11β-HSD1 expression can be increased by glucocorticoids, by pro-inflammatory Cytokines and in ageing in other tissues and this is also likely to be the case in the heart.

11β-hydroxysteroid dehydrogenase (11β-HSD) catalyses the intracellular interconversion of the glucocorticoids cortisol and corticosterone with their inert 11-keto forms (cortisone and 11-dehydrocorticosterone (11-DHC), respectively). 11β-HSD type 2 (11β-HSD2) is a high-affinity (nanomolar *K*M), low-capacity NAD-dependent dehydrogenase (Albiston *et al.* 1994, Brown *et al.* 1996) that inactivates the glucocorticoids (Fig. 1). 11β-HSD1 shares less than 30% homology with 11β-HSD2 (Lakshmi & Monder 1988, Agarwal *et al.* 1989) and is more widely distributed than 11β-HSD2. Under normal conditions, it is co-expressed at the luminal border of the endoplasmic reticulum alongside hexose-6-phosphate dehydrogenase (H6PDH) (Atanasov *et al.* 2008) that provides the co-substrate NADPH (Bujalska *et al.* 2005). This drives the oxo-reductase activity of 11β-HSD1, re-activating the glucocorticoids (Atanasov *et al.* 2004, Bujalska *et al.* 2005, Lavery *et al.* 2006, Chapman *et al.* 2013*a*) (Fig. 1). 11β-HSD1 has low affinity for glucocorticoids (micromolar *K*M) relative to 11β-HSD2, but where H6PDH is genetically deleted (Lavery *et al.* 2006) or where cells are disrupted so that NADPH cannot be generated in close proximity to 11β-HSD1, it switches from predominantly oxo-reductase to dehydrogenase activity and this inactivates rather than regenerates glucocorticoids (Atanasov *et al.* 2004). Activity of 11β-HSD1 (largely in the liver) and 11β-HSD2 (largely in the kidney) determines active and keto-isoform concentrations in the systemic circulation, but the variability in the activity of 11β-HSD enzymes at the level of target cells adds a cell-specific dimension to the control of steroid action (Chapman *et al.* 2013*a*). To date, there has been relatively little focus on the capacity of the myocardium or of its component cells to metabolise corticosteroids.

## 11β-HSD2 and MR activation in the heart

In mineralocorticoid target tissues, 11β-HSD2 is expressed alongside the MR, where its activity reduces the availability of glucocorticoid, permitting aldosterone to compete for binding to the MR (Edwards *et al.* 1988, Funder *et al.* 1988). The clinical efficacy of MR antagonists after MI (Pitt *et al.* 2003) and in heart failure (Pitt *et al.* 1999, Zannad *et al.* 2011, 2012) are attributed, at least in part, to blockade of MR activation in the heart. A recent preclinical study in adrenalectomised rats has shown that mineralocorticoids regulate cardiac electrical function through the activation of MR (Cruz-Topete *et al.* 2016). However, the weight of evidence suggests that there is normally little or no 11β-HSD2 activity in the heart (Walker *et al.* 1991, Whorwood *et al.* 1992), except perhaps in vascular endothelium (Walker *et al.* 1991, Smith *et al.* 1996). This was confirmed in a recent *in vivo* study on patients under­going diagnostic coronary angiography that failed to find any evidence for inactivation of the stable isotope tracer 9,11,12,12-[2H]4-cortisol across the human heart (Iqbal *et al.* 2014). Furthermore, infusion of the MR antagonist canrenoate in the same patients resulted in the elevation of cortisol collected from the coronary sinus. These data support the view that the cardiac MR is normally occupied by glucocorticoid, rather than by aldosterone. However, this situation may change in pathological conditions, including hypoxia (Klusonova *et al.* 2009), in which there is evidence for enhancement of 11β-HSD2 activity and expression (Chai *et al.* 2010, Chapman *et al.* 2013*a*), that may allow aldosterone to compete for binding to the MR. Selective overexpression of 11β-HSD2 in cardiomyocytes leads to MR-dependent hypertrophy and fibrosis in mice (Qin *et al.* 2003), showing potential for a role in cardiac pathology. Global 11β-HSD2 knockout mice have cardiac hypertrophy and fibrosis but, as these mice are also hypertensive (Kotelevtsev *et al.* 1999), any phenotype that is specifically due to lack of 11β-HSD2 in the heart has proved impossible to dissect. Generation of mice with targeted 11β-HSD2 deletion in cardiomyocytes and other cardiac cells would provide a more definitive answer regarding the role of dehydrogenase activity in cardiac physiology and pathology.

## Glucocorticoid regeneration by 11β-HSD1 in the heart

11β-HSD1 is more widely expressed in the body than 11β-HSD2, but tissue expression of 11β-HSD1 is often reported to be low. This is also the case in the heart or at least in whole ventricular homogenates (Walker *et al.* 1991, White *et al.* 2016). However, immunoreactive 11β-HSD1 can be localised to cardiomyocytes, to interstitial and adventitial fibroblasts (Brereton *et al.* 2001) and to vascular smooth muscle in murine heart (Fig. 1) (McSweeney 2010). We have found that targeted deletion of 11β-HSD1 in cardiomyocytes and vascular smooth muscle cells leads to a significant reduction in *Hsd11b1* gene expression in the mouse heart, confirming its expression in these cells (White *et al.* 2016). Although endothelial cells in culture are reported to have 11β-HSD1 activity (Brem *et al.* 1998), previous studies have concluded that the 11β-HSD enzyme in aortic endothelial cells is primarily a dehydrogenase, with oxo-reductase activity limited to the smooth muscle cell compartment of the vascular wall (Walker *et al.* 1991, Dover *et al.* 2007). Immunostaining suggests that this is also the case in the coronary vessels (Fig. 1). Fibroblasts are among the most abundant cells in the heart, and transcriptomic analysis has revealed that expression of *Hsd11b1* is high in cardiac fibroblasts relative to fibroblasts elsewhere in the body (Furtado *et al.* 2014), consistent with a potentially important role here.

**Figure 2:**
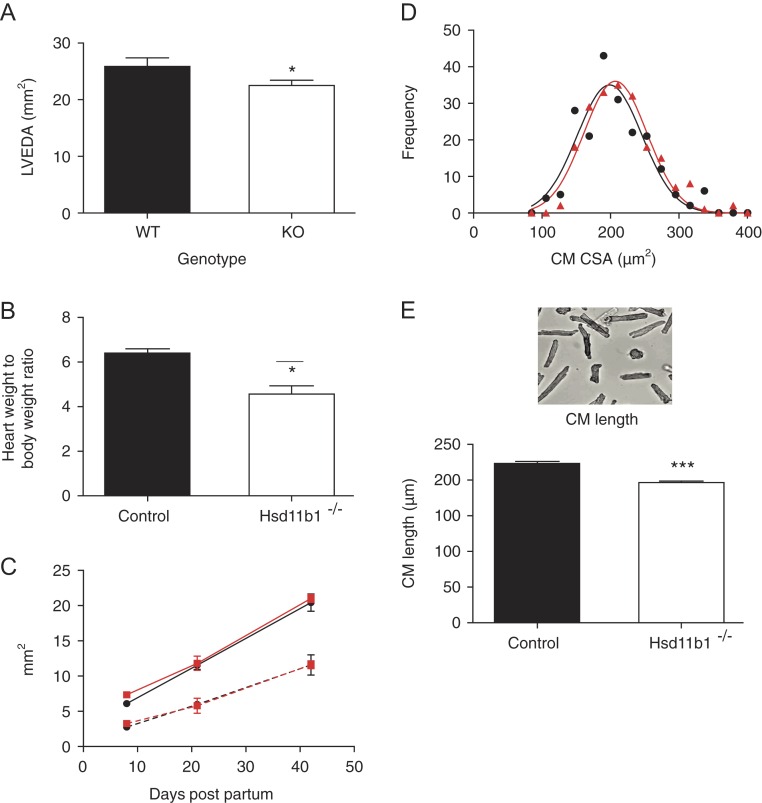
Hearts from adult mice with global 11β-HSD1 deficiency are hypomorphic and have shorter cardiomyocytes. Ultrasound analysis (A) reveals a significant reduction in left ventricular dimensions in 12-week-old *Hsd11b1*^−/−^ (open bars) compared with control wild-type mice (filled bars). Reduction in heart size was confirmed when post-mortem heart weight was compared with body weight (B). LV dimensions (left ventricular end-diastolic area, solid red (*Hsd11b1*^−/−^) and black (control wild type), and left ventricular end-systolic area, hatched red (*Hsd11b1*^−/−^) and black (control wild type)) were not influenced by the loss of 11β-HSD1 from late gestation until 6 weeks of age (C). In adult hearts, there was no effect of 11β-HSD1 deficiency (red) on the distribution of cardiomyocyte cross-sectional area (D), but the length of individual cardiomyocytes (E) isolated from *Hsd11b1*^−/−^ hearts was significantly less than that isolated from control wild-type hearts (black).*P < 0.05, ***P < 0.005, *n* = 5–8.

Myocardial expression of H6PDH (Brereton *et al.* 2001, Gomez-Sanchez *et al.* 2008), and H6PDH immunoreactivity in cells identified as fibrocytes (Gomez-Sanchez *et al.* 2008), suggests that NADPH will be available to support oxo-reductase activity in the heart. However, conversion of 11-DHC to corticosterone is comparatively low in whole ventricular homogenate from mice and rats (Walker *et al.* 1991, Sheppard & Autelitano 2002). Similarly, although *HSD11B1* mRNA is present in human heart (Chai *et al.* 2010), cortisol is not significantly regenerated from cortisone infused *in vivo* into the hearts of patients undergoing elective coronary angiography (Iqbal *et al.* 2014). Thus, although there is plentiful evidence that myocardial cells express 11β-HSD1, reactivation of glucocorticoid is apparently limited under physiological conditions. Nevertheless, our phenotyping studies in mice with genetically induced 11β-HSD1 deficiency show that 11β-HSD1 activity has a role in determining normal postnatal growth of the heart (Fig. 2 and below), as well as Ca^2+^ handling and diastolic function (White 2016).

## 11β-HSD1 in postnatal growth of the myocardium

GR activation during a prenatal surge in plasma glucocorticoid is essential for the structural and functional maturation of the heart before birth (Rog-Zielinska *et al.* 2014, 2015), and consequently, genetic deletion of GR from the heart and smooth muscle results in increased perinatal mortality (Rog-Zielinska *et al.* 2013, 2014). 11β-HSD1 is not expressed to any extent in the mouse heart in the prenatal period (Speirs *et al.* 2004), and neither global (Kotelevtsev *et al.* 1997) nor cardiomyocyte and vascular smooth muscle cell-specific (White *et al.* 2016) deletion of *Hsd11b1* has any influence on perinatal mortality. However, a role for 11β-HSD1 in the maturation of the postnatal heart is indicated by our observation that hearts from adult mice with global deficiency of 11β-HSD1 are smaller than those of wild-type mice (McSweeney 2010, McSweeney *et al.* 2010; Fig. 2A). Cardiomyocyte GR activation in the neonatal period promotes the switch from proliferative to hypertrophic growth (Richardson *et al.* 2016). MR activation in the same period promotes proliferation (Feng *et al.* 2013). Proliferative neonatal cardiomyocytes can reactivate glucocorticoids through 11β-HSD1 oxo-reductase activity during the period that they retain proliferative capacity (Lister *et al.* 2006). However, the hearts of globally 11β-HSD1-deficient mice have normal ventricular dimensions from E18.5 to at least P40 (Fig. 2B). Mice with targeted deletion of 11β-HSD1 in cardiomyocytes and vascular smooth muscle also have phenotypically normal hearts (White *et al.* 2016). Therefore, loss of cardiomyocyte corticosteroid receptor activation in the perinatal period does not account for the smaller size of hearts in adult 11β-HSD1 mice. Interestin­gly, further analysis revealed that although cardiomyocyte cross-sectional area is normal in adult hearts of 11β-HSD1-deficient mice (Fig. 2C), cardiomyocyte length is reduced (Fig. 2D; White 2016). Cardiomyocytes are influenced by physical interactions and paracrine signalling from other cells in their environment. Blood volume and blood pressure are not modified in mice with global 11β-HSD1 deficiency, so altered *in vivo* haemodynamic stress is not likely to underlie the reduction in cardiomyocyte length (White 2016). Further investigation is required to find whether the loss of 11β-HSD1 in other myocardial cells, for example, resident macrophages or fibroblasts can indirectly influence cardiomyocyte size during postnatal growth. In terms of clinical relevance, it is notable that a single nucleotide polymorphism in the *HSD11B1* gene is also associated with reduced left ventricular mass (Rahman *et al.* 2011), although the underlying mechanism was not investigated.

## 11β-HSD1 and myocardial pathology

Although cellular 11β-HSD1 expression and activity may normally be low in the heart, both can change rapidly in response to external stimuli (reviewed in Chapman *et al.* 2013*b*). Glucocorticoid itself promotes 11β-HSD1 expression (Sai *et al.* 2008, Morgan *et al.* 2014), and there is good evidence that 11β-HSD1 is the major regulator of the tissue-specific effects of circulating glucocorticoid excess (Morgan *et al.* 2014). The pro-inflammatory cytokines IL-1 and TNF-α are also key activators of 11β-HSD1 gene expression, notably in fibroblasts (Hardy *et al.* 2006, Stegk *et al*. 2009, Ahasan *et al.* 2012, Chapman *et al.* 2013*b*, Esteves *et al.* 2014). These cytokines can act alone or synergistically with glucocorticoid (Kaur *et al.* 2010, Ahasan *et al.* 2012), to effect an increase in the capacity for intracellular glucocorticoid regeneration by 11β-HSD1. After trauma or injury, pro-inflammatory conditions that potently increase HPA axis activity (and thereby plasma 11-DHC/cortisone levels (Harris *et al.* 2001)), increased 11β-HSD1 activity is likely to be a significant contributor to intracellular glucocorticoid levels and thus GR and MR activation. The generation of mice lacking 11β-HSD1 (Kotelevtsev *et al.* 1997, Holmes *et al.* 2001) and more recently, the development of specific 11β-HSD1 inhibitors (Hughes *et al.* 2008, Wheelan *et al.* 2014, Sooy *et al.* 2015), have provided the opportunity to identify such roles for amplification of intracellular glucocorticoid signalling by 11β-HSD1 in cardiac pathophysiology.

## 11β-HSD1, MI and heart failure

MI occurs most commonly after the formation of a clot on a ruptured atherosclerotic plaque, leading to ischaemic cardiomyocyte death in the area served by the affected coronary artery. As the adult heart has only very limited ability to regenerate, MI is followed by a period of wound healing that results in the formation of a scar to maintain myocardial integrity (Video 1; Frangogiannis 2014). The extent of cardiomyocyte loss during ischaemia and expansion of injury during infarct repair are critical determinants of subsequent structural remodelling and functional deterioration leading to heart failure (Di Bella *et al.* 2013).

Video 1 Coronal view of mouse heart collected 7 days after induction of myocardial infarction and imaged by optical projection tomography, as we have described in (Zhao *et al.* 2015). Progressive optical sectioning through the heart reveals the extensive, thinned infarct area of the left ventricle. In 11β-HSD1 deficient mice the length of the infarcted area is reduced and the ventricle wall is less thinned (McSweeney *et al.* 2010, White 2016). View Video 1 at http://movie-usa.glencoesoftware.com/video/10.1530/JME-16-0128/video-1
Download Video 1

In a preclinical model of atherosclerosis in ApoE-deficient mice, pharmacological inhibition (Hermanowski-Vosatka *et al.* 2005), or genetic ablation (Garcia *et al.* 2013, Kipari *et al.* 2013), of 11β-HSD1 is associated with reduction in plaque size independently of changes in lipid availability. Plaque size reduction in ApoE/11β-HSD1 double knockout mice was attributed to loss of 11β-HSD1 in bone marrow-derived cells (Kipari *et al.* 2013). These cells are recruited to the arterial wall during plaque formation, where they accumulate lipid and contribute to the determination of plaque stability. Neutrophils, monocyte/macrophages and T cells express 11β-HSD1 (Zhang & Daynes 2007, Zhang *et al.* 2005), and expression is increased during their mobilisation and activation (reviewed in Chapman *et al.* 2013*b*). Intracellular generation of glucocorticoid in monocyte/macrophages and neutrophils regulates their recruitment to sites of inflammation (Tiganescu *et al.* 2011), phagocytic potential (Speirs *et al.* 2004) and the release of pro-inflammatory molecules (Zhang & Daynes 2007). 11β-HSD1 inhibition may therefore be of therapeutic benefit in preventing the development of complex plaques that are vulnerable to rupture causing MI.

**Figure 3:**
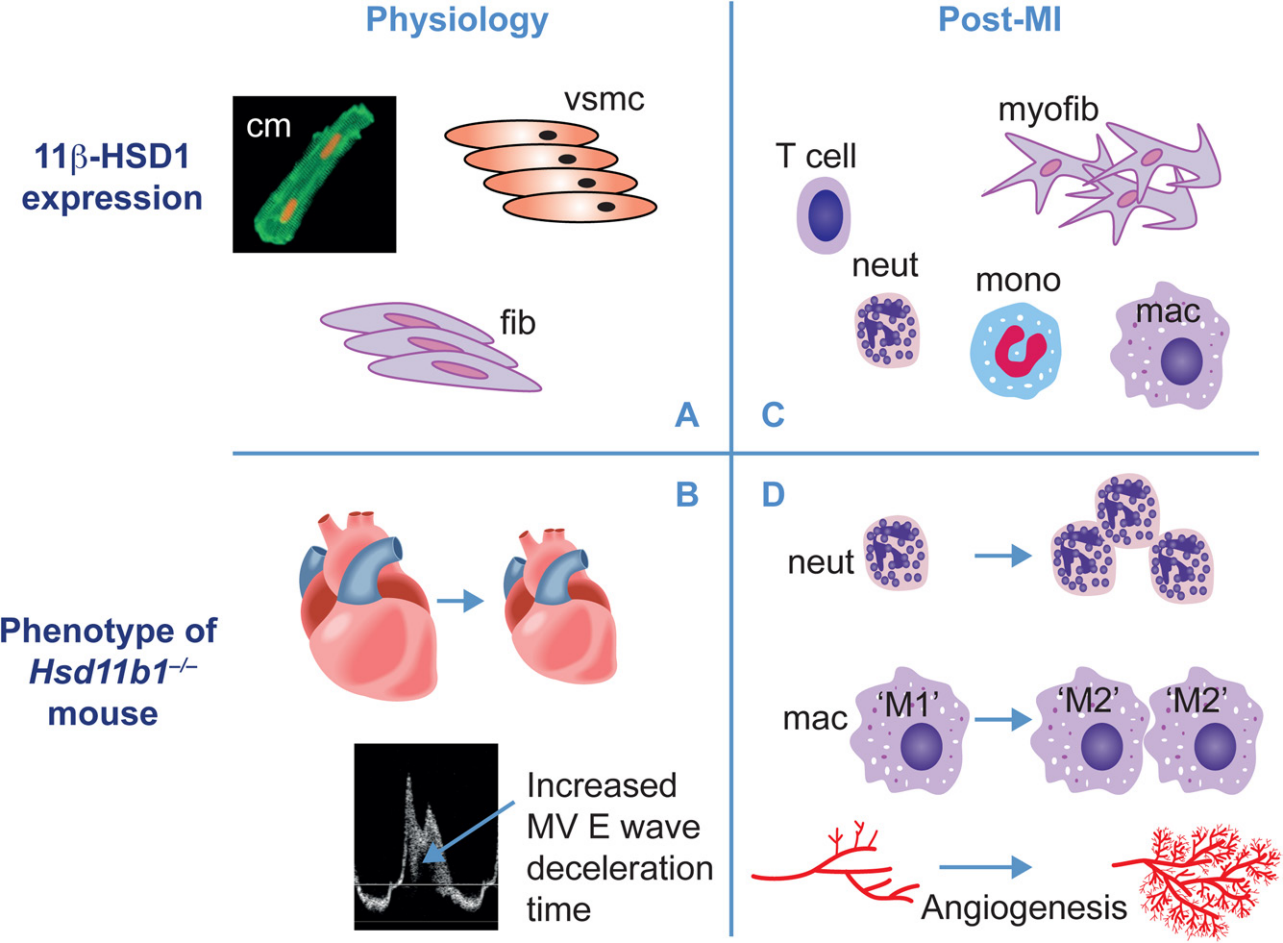
Distribution and actions of 11β-HSD1 in physiology and post-MI. In the healthy heart (panel A), 11β-HSD1 is expressed in cardiomyocytes (cm), vascular smooth muscle (VSMC) and fibroblasts (fib). Deletion of 11β-HSD1 (panel B) results in smaller adult hearts and mild diastolic dysfunction (identified by increased deceleration time on Doppler mitral valve (MV) ultrasound). After MI (panel C), 11β-HSD1 is additionally expressed in myofibroblasts (myofib) and recruited inflammatory cells (T cells, neutrophils (neut), monocyte (mono) and macrophages (mac)). 11β-HSD1 deficiency after MI (panel D) results in increased early neutrophil (neut) recruitment, increased acquisition of pro-repair ‘M2’ macrophage (mac) phenotype, relative to pro-inflammatory ‘M1’ macrophage phenotype and increased angiogenesis, leading to increased vessel density.

Mice with global deficiency of 11β-HSD1 also have reduced structural and functional remodelling after MI, induced by coronary artery ligation, and do not go on to develop heart failure (McSweeney *et al.* 2010, White *et al.* 2016). Thus, 11β-HSD1 availability has an impact not only on the development of coronary artery disease that leads to MI but also on the response of the myocardium after MI. Increased plasma corticosterone, derived from HPA axis activation in the immediate post-infarct period, reduces cardiomyocyte death and infarct size and there is evidence supporting roles for both GR (Hafezi-Moghadam *et al.* 2002) and MR (Mihailidou *et al.* 2009, Fraccarollo *et al.* 2011) in determining this outcome. However, in 11β-HSD1-deficient mice, plasma corticosterone is increased to the same extent after MI as in wild-type mice and infarct injury is unchanged (McSweeney *et al.* 2010). Therefore, differences in the immediate response to MI do not underlie the subsequent reduction in cardiac remodelling in the absence of 11β-HSD1. However, peri-infarct angiogenesis is enhanced in 11β-HSD1-deficient mice during repair (Small *et al.* 2005), and this is associated with reduced infarct expansion in this period and with eventual scar size (McSweeney *et al.* 2010). Glucocorticoids suppress angiogenesis, and prevention of intracellular glucocorticoid regeneration in 11β-HSD1-deficient mice promotes angiogenesis in different *in vitro* and *in vivo* experimental models (Small *et al.* 2005). Smooth muscle cells are the main site of intravascular 11β-HSD1 activity (Hadoke *et al.* 2013), but targeted deletion of *Hsd11b1* in cardiomyocytes and vascular smooth muscle cells failed to enhance angiogenesis or improve outcome after MI (White *et al.* 2016). Engagement of extra-vascular pro-angiogenic mechanisms is therefore indicated. Increased angiogenesis in 11β-HSD1-deficient mice after MI is preceded by increased content of reparative ‘M2’ macrophages during the proliferative phase of wound healing (McSweeney *et al.* 2010). These ‘M2’ macrophages are essential for infarct repair (Shiraishi *et al.* 2016) and can release growth factors and pro-angiogenic mediators that increase neovascularisation during wound healing, as well as for promoting the formation of a mature collagen scar. Enhancement of macrophage polarisation in mice lacking 11β-HSD1 is therefore likely to be a key mechanism underlying improved outcome after MI (Fig. 3). Increased ‘M2’ polarisation of macrophages could occur due to loss of 11β-HSD1 in the inflammatory cells themselves, as proposed in atherosclerosis (Kipari *et al.* 2013), resulting in the modification of inflammatory cell recruitment and phenotype (Frangogiannis 2014). Macrophage MR knockout (Usher *et al.* 2010), or GR activation (Gratchev *et al.* 2008), results in polarisation towards an ‘M2’ phenotype, and MR antagonists increase ‘M2’ macrophage polarisation in the heart when given after MI (Fraccarollo *et al.* 2008). Loss of glucocorticoid regenerative capacity in macrophages could therefore conceivably underlie changes in the activation status by preventing the activation of MR. However, promotion of peri-infarct angiogenesis after MI is enhanced when GR is blocked (Small *et al.* 2005), supporting a more indirect mechanism that involves GR, rather than MR activation.

Neutrophil recruitment is increased in the hearts of 11β-HSD1-deficient mice after the induction of MI (McSweeney *et al.* 2010) (Fig. 3). This results at least in part from loss of 11β-HSD1 in neutrophils themselves, where its activity restrains recruitment to inflamed tissues through modulation of adhesion molecule availability at the cell surface (Cavalcanti *et al.* 2006, Tiganescu *et al.* 2011, Coutinho *et al.* 2016). Loss of 11β-HSD1 activity in the heart itself is also likely to contribute. Expression of neutrophil chemoattractants, including CXCL5/LIX (Smith & Herschman 1995), IL-6 (Hardy *et al.* 2006) and CXCL2/MIP2α (Uhlenhaut *et al.* 2013) is repressed by glucocorticoid, and these chemoattractants are expressed by cells in the heart that express 11β-HSD1, including fibroblasts. Mast cell degranulation after myocardial injury also enhances neutrophil recruitment, and as degranulation is increased in the absence of 11β-HSD1 (Coutinho *et al.* 2013), these cells may also play a role in increasing neutrophil recruitment in 11β-HSD1-deficient mice. In the post-infarct heart, neutrophils phagocytose necrotic myocytes and break down the intracellular matrix during the initial inflammatory phase of repair. However, they also promote the transition to resolution by releasing gelatinase-associated lipocalin (Horckmans *et al.* 2016). Efferocytosis, or macrophage engulfment of apoptotic cells, including neutrophils (Wan *et al.* 2013), will also enhance the acquisition of an ‘M2’ phenotype. Early enhancement of neutrophil recruitment may thus be an essential prerequisite for the promotion of repair in the mice lacking 11β-HSD1.

Several lines of evidence point to cardiac fibroblasts, particularly their high expression of 11β-HSD1 (Furtado *et al.* 2014), as a key site for intracellular regeneration of glucocorticoid in the healing myocardial infarct (Chen & Frangogiannis 2013). 11β-HSD1 activity in stromal cells elsewhere, including synovial fibroblasts, is promoted by the action of corticosteroids and inflammatory mediators (Hardy *et al.* 2006, Ahasan *et al.* 2012). In the skin, 11β-HSD1 is active in dermal fibroblasts (Tiganescu *et al.* 2011) and global 11β-HSD1 knockout or pharmacological inhibition promotes skin wound healing (Tiganescu *et al.* 2013). Fibroblasts are abundant in the heart and have multiple potential roles during myocardial inflammation and repair that follows MI (Porter & Turner 2009). In addition to the production of collagen and other matrix proteins that determine scar integrity, they secrete molecules, including cyto­kines, prostaglandins (Shinde & Frangogiannis 2014, Fernando *et al.* 2015, Turner 2016) and microRNA (Fang & Yeh 2015), capable of regulating inflammation (Porter & Turner 2009, Chen & Frangogiannis 2013, van Nieuwenhoven & Turner 2013), Treg recruitment (Frangogiannis 2014), angiogenesis (Newman *et al.* 2011), cardiogenesis (Furtado *et al.* 2014) and hypertrophy (Abonnenc *et al.* 2013, Cartledge *et al.* 2015). The role of cardiac fibroblast 11β-HSD1 in regulating the cellular secretome and on the response to MI and other pathological challenges merits further investigation.

## Therapeutic potential of 11β-HSD1 inhibition in myocardial disease

Pharmacological inhibitors of 11β-HSD1 have already been developed for use in metabolic disease and for prevention of cognitive decline (Anderson & Walker 2013, Sooy *et al.* 2015). Survival after MI is increasing, thanks to prompt and efficient intervention to restore perfusion to the ischaemic myocardium (BHF Heart Stats, 2015). However, patients survive with damage to their myocardium that spreads to the peri-infarct area during wound healing. Thus, although the introduction of coronary revascularisation has been associated with a reduction in early post-MI deaths, there has been an increase in the 5-year incidence of HF (Velagaleti *et al.* 2008). Preclinical data are highly supportive of the therapeutic potential of 11β-HSD1 inhibitors in the prevention of atherosclerosis (Hermanowski-Vosatka *et al.* 2005, Garcia *et al.* 2013, Kipari *et al.* 2013), as well as acutely following MI, to prevent the infarct expansion that promotes the development of heart failure (McSweeney *et al.* 2010, White *et al.* 2016). Ventricular hypertrophy is a feature of the adaptive ventricular remodelling that follows MI and in pressure overload associated with hypertension. Gordon and coworkers (2014) showed that an inhibitor of 11β-HSD1 was able to reverse established hypertrophy and associated dysfunction in a mouse model of perfusion deficit-induced cardiac remodelling. Interestingly, this outcome was independent of any influence on neovascularisation or perfusion. Inhibition of 11β-HSD1 may therefore have additional benefits on post-MI remodelling beyond the prevention of infarct expansion. In clinical trials of 11β-HSD1 inhibitors in diabetes, improvement in blood glucose and other metabolic end points failed to reach significance when compared with existing therapies, but the evidence for a modest benefit was highly consistent (Anderson & Walker 2013). Such benefits in metabolic outcomes are likely to be advantageous in patients with cardiovascular disease. Together, these data demonstrate the potential for ‘repurposing’ of 11β-HSD1 inhibitors already developed for clinical use in other therapeutic areas. None of the trials of these inhibitors in diabetes have raised any question on the effectiveness of target engagement or on toxicology. Improved understanding of the cellular mechanisms through which 11β-HSD1 inactivation enhances angiogenesis prevents infarct expansion, and adverse ventricular remodelling will help to justify the clinical development of 11β-HSD1 inhibitors for MI therapy, as well as providing biomarkers to monitor the effects of 11β-HSD1 inhibition.

MR antagonists are already used effectively in the treatment of heart failure (Pitt *et al.* 1999, Zannad *et al.* 2011, 2012), by preventing MR activation in cardiomyocytes (Fraccarollo *et al.* 2011) and in macrophages (Fraccarollo *et al.* 2008). Preclinical studies have revealed the potential benefits of blocking GR in cardiovascular disease (Small *et al.* 2005, Oakley & Cidlowski 2015, Richardson *et al.* 2016), but essential physiological roles of GR activation preclude the use of GR antagonists *in vivo*. With their distinct profile of action, 11β-HSD1 inhibitors offer an alternative means of regulating GR and MR activation in specific cells. 11β-HSD1 inhibitors might provide an alternative to MR antagonists in patients who do not tolerate the effects of MR antagonism on K^+^ handling (Pitt *et al.* 2008). Alternatively, they may have additional or complementary benefits when combined with MR antagonists, and this should be tested in models of cardiovascular disease.

In this review, we have considered 11β-HSD1 in the context of its capacity for intracellular regeneration of active glucocorticoids from inert metabolites. However, 11β-HSD1 has other actions that may contribute to the physiological and pathophysiological outcomes reported previously. 11β-HSD1 is a multifunctional carbonyl reductase that also converts 11- and 7-oxosterols into the respective 7-hydroxylated forms and also catalyses the reduction of non-steroidal xenobiotics (Mitic *et al.* 2013*b*, Odermatt & Klusonova 2015). Studies with selective inhibitors and genetically modified mice provide further evidence for the effects of 11β-HSD1 in the regulation of cellular cholesterol flux and on bile acid homeostasis (Mitic *et al.* 2013*a*). Oxysterols influence vascular function (Mitic *et al.* 2013*a*), and modulation of genes involved in cellular cholesterol flux is associated with disease severity in patients after MI (Suresh *et al.* 2014). These mechanisms may therefore be engaged alongside prevention of intracellular glucocorticoid regeneration when 11β-HSD1 is genetically depleted or inhibited and could contribute to any beneficial effects of drug intervention in cardiovascular pathology.

## Conclusion

Studies over the past three decades have demonstrated roles for the 11β-HSD enzymes in metabolic, neurological and inflammatory diseases, but roles in the heart have been relatively unexplored. Recent studies have shown that 11β-HSD1 is expressed in myocardial cells, including notably cardiac fibroblasts. Global 11β-HSD1 deficiency has effects on cardiac growth and physiological function that are more subtle than those associated with GR deletion (Oakley & Cidlowski 2015, Richardson *et al.* 2016), or adrenalectomy (Cruz-Topete *et al.* 2016), but nevertheless of physiological relevance. Importantly, genetic or pharmacological depletion of 11β-HSD1 also prevents adverse cardiac remodelling and the development of heart failure after MI (McSweeney *et al.* 2010, Gordon *et al.* 2014, White *et al.* 2016). In the skin, 11β-HSD1 expression is increased during ageing (Tiganescu *et al.* 2011) and 11β-HSD1 inhibition prevents age-induced structural defects (Tiganescu *et al.* 2013). If 11β-HSD1 expression is similarly increased in the heart, the influence of 11β-HSD1 on myocardial physiology and pathophysiology is likely to be even more pronounced in the aged heart. Novel mass spectrometry imaging has recently permitted accurate localisation of 11β-HSD1 ‘intracrinology’ in sections of mouse adrenal gland and brain (Cobice *et al.* 2013). Application of mass spectrometry imaging to the heart will be of significant value to understand cellular 11β-HSD1 activity and how it is changed in ageing and disease. Whether the key intracellular mechanisms of 11β-HSD1 are dependent or independent of glucocorti­coid regeneration, preclinical studies have started to reveal novel therapeutic potential for drugs that inhibit 11β-HSD1. Given that 11β-HSD1 expression and activity can be rapidly increased by inflammatory mediators and by glucocorticoids, there are likely to be further applications for these drugs in cardiovascular disease, as well as in preventing the deterioration of cardiac function associated with chronic stress, Cushing’s disease and glucocorticoid therapy (Souverein *et al.* 2004, Wei *et al.* 2004, Peppa *et al.* 2009, Kumari *et al.* 2011, Shibusawa *et al.* 2015, Frustaci *et al.* 2016).

## Declaration of interest

The authors declare that there is no conflict of interest that could be perceived as prejudicing the impartiality of this review.

## Funding

Work in the authors’ lab is supported by the British Heart Foundation (FS/09/053 and FS/12/30002), the Wellcome Trust (083184) and the British Heart Foundation Centre of Research Excellence.
